# Dating psychological violence among adolescents in a metropolitan region of southeastern Brazil: a school-based study

**DOI:** 10.3389/fpubh.2026.1870784

**Published:** 2026-08-27

**Authors:** Isaura Barros Alves Pinto, Franciele Marabotti Costa Leite

**Affiliations:** 1 National Immunization Program Department (DPNI), Ministry of Health (Brazil), Brasília, Brazil; 2 Postgraduate Program in Collective Health, Hésio Cordeiro Institute of Social Medicine (IMS), State University of Rio de Janeiro (UERJ), Rio de Janeiro, Brazil; 3 Postgraduate Program in Collective Health, Federal University of Espírito Santo (UFES), Vitória, Brazil

**Keywords:** adolescent health, dating violence, epidemiology, intimate partner violence, violence

## Abstract

**Objective:**

To analyze the prevalence of dating psychological violence among adolescents in the Metropolitan Region of the State of Espírito Santo (ES), Brazil, and its association with socioeconomic and behavioral characteristics and violence experiences.

**Methods:**

A cross-sectional and analytical study was conducted with 1,238 adolescents aged 14–19, enrolled in public and private schools in the Greater Vitória Metropolitan Region (ES), Brazil, who were in a romantic relationship at the time of the research. Sampling was by cluster and stratification. The outcome variable was dating psychological violence (yes/no), and the independent variables included sociodemographic characteristics, risk behaviors, and previous violence experiences. Absolute and relative frequencies were calculated. Bivariate analysis used Pearson’s chi-square test (*χ*^2^) with a 95% confidence interval. Logistic regression was applied to estimate crude and adjusted associations. Variables with *p* ≤ 0.20 were included in the multivariate model, while those with *p* ≤ 0.05 remained in the model.

**Results:**

We observed that 18.6% (95% CI: 16.5–20.8) of the participants reported having suffered dating psychological violence. The prevalence was 21.7% among girls and 13.1% among boys. Furthermore, higher frequencies of the problem were observed among non-heterosexual adolescents, those who had had sexual intercourse at some point in their lives, those who smoked cigarettes, as well as those who had suffered bullying (*p* < 0.05).

**Conclusion:**

The findings show that early romantic relationships can involve abusive behaviors linked to social vulnerability and prior violence, highlighting the need for education on recognizing abuse and fostering healthy, respectful relationships.

## 1 Introduction

Adolescence is a stage of intense change, in which friendships and romantic relationships take center stage and family dynamics undergo adjustments. In this context, youth behaviors are influenced by a combination of individual, social, and intergenerational factors, which can make adolescents more susceptible to incorporating norms from the groups to which they belong, including those that end up normalizing situations of violence, such as psychological violence (1).

Dating violence (DV) is understood as any form of abuse perpetrated by an intimate partner, encompassing physical, emotional, and sexual dimensions (2). Among these manifestations, psychological violence plays a central role, involving control, blackmail, intimidation, humiliation, and manipulation strategies that compromise the adolescent’s autonomy and emotional well-being. Although often invisible, this type of abuse is a severe violation of rights and profoundly impacts the victim’s mental health (3).

Evidence indicates that this type of violence can occur even in informal relationships or in early stages, such as first dates, and affects adolescents of different identities and sexual orientations (4). Epidemiological data show the magnitude of dating psychological violence among adolescents. In a national study conducted with Brazilian students, Veríssimo et al. (5) identified that 81.8% of adolescents reported having experienced some episode of psychological violence from their partner.

Significantly, this type of violence is associated with significant impacts on mental health, such as depressive symptoms, post-traumatic stress disorder, and a higher likelihood of substance use, as pointed out by the CDC (6). Furthermore, the normalization of these practices, often sustained by social norms and gender inequalities, prevents adolescents from recognizing such behaviors as abusive and seeking support or reporting them (1).

Although the national literature points to associations between sociodemographic factors and psychological dating violence, studies specifically focusing on Brazilian adolescents remain scarce (7). A recent systematic review identified that national research has primarily investigated factors associated with perpetration and victimization, prevalence patterns, and coping strategies (7). Alcohol consumption, characteristics such as gender and non-heterosexual sexual orientation, as well as prior exposure to violence, rank among the main predictors of this outcome in the country’s young population (8, 9). Differences between boys and girls in victimization and perpetration patterns have also been documented, with heterogeneous profiles reinforcing the importance of sex-stratified analyses (9).

Given this situation, this study aims to analyze the prevalence of dating psychological violence (DPV) among adolescents in the Metropolitan Region of the State of Espírito Santo, and its association with socioeconomic and behavioral characteristics and experiences of violence.

## 2 Methods

### 2.1 Study design and context

This cross-sectional, analytical study employed stratified cluster sampling in public and private schools in Espírito Santo, Brazil, in municipalities of the Greater Vitória Metropolitan Region (RMGV). RMGV comprises Cariacica, Fundão, Guarapari, Serra, Viana, Vila Velha, and Vitória. This region is home to approximately 1,880,828 inhabitants, distributed across an area of 2,331 km^2^ (10).

### 2.2 Sample

The target population of the study was high school students, aged 14–19, regularly enrolled in public or private schools in the RMGV, who participated after their parents/guardians accepted the Informed Consent Form and the adolescents signed the Informed Assent Form and were informed about the objectives of the research and its potential risks and benefits.

For the present study, only adolescents who had some relationship at the time of the interview were selected, totaling a sample of 1,238 adolescents.

### 2.3 Sample calculation

The sampling used a 95% confidence level and a maximum error of 5%. The maximum error was 10% for each municipality stratum with a minimum sample size of 4,416 students.

### 2.4 Data collection

Before the data collection phase, the selected schools were invited by email to participate in a seminar presenting the research, and, subsequently, contact was made by email/telephone to schedule data collection. Notably, the team was recruited and trained, and a pilot test was conducted in February before the fieldwork. Data were collected from March to December 2023, using tablets, where the adolescents self-answered the structured questionnaire in the REDCAP (Research Electronic Data Capture) system.

### 2.5 Study variables

#### 2.5.1 Outcome

The outcome variable in this study was dating psychological violence (yes/no), understood as an affective relationship where the partner diminished the victim’s self-esteem, threatened, coerced, humiliated, constantly monitored, continuously stalked, insulted, blackmailed, or used any other means that caused harm to psychological health.

#### 2.5.2 Independent variables

The independent variables analyzed were sociodemographic: gender (female/male); age range (≤15 years/≥16 years); cisgender (yes/no); heterosexual (yes/no); ethnicity/skin color (white/black/brown); economic classification was measured using the ABEP Economic Classification Criteria of the Brazilian Association of Research Companies, which classify people into classes A/B/C/D and E; work/job (no/yes) and religion (none/Catholic/Evangelical/other). Behavioral variables: sexual intercourse in lifetime (no/yes); current cigarette use (no/yes); current drug use (no/yes) and current alcohol use (no/yes). Variables related to violence experiences, namely, has suffered cyberbullying in lifetime (no/yes); has perpetrated cyberbullying in lifetime (no/yes); has suffered bullying in lifetime (no/yes); has perpetrated bullying in lifetime (no/yes) (32).

### 2.6 Data analysis

**Figure 1:**
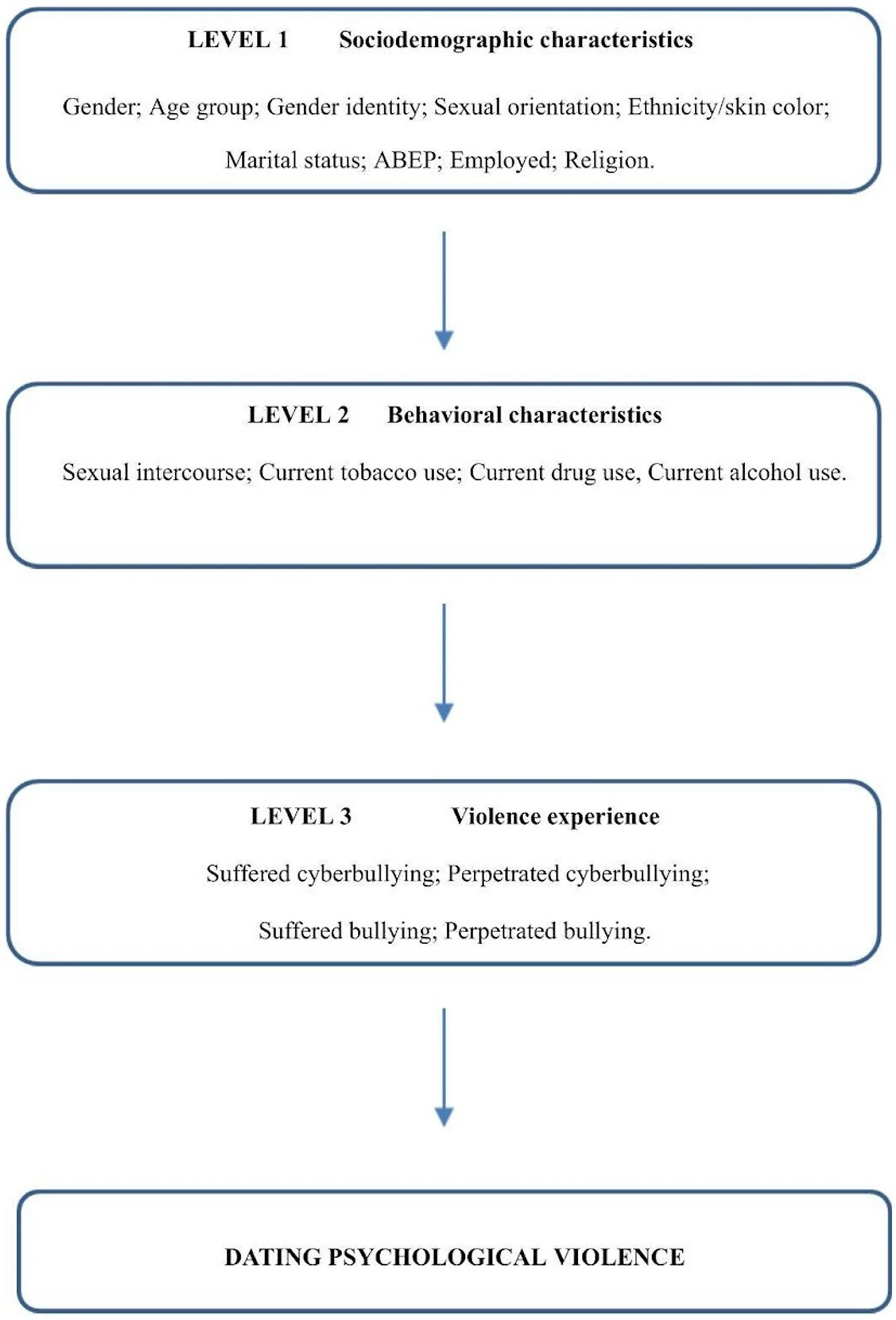
Level-based analysis model – flowchart.

We calculated absolute and relative frequencies for the descriptive statistical analyses. In the bivariate analysis, we adopted Pearson’s Chi-square test (*χ*^2^) as per assumptions, with its respective 95% confidence intervals. We performed logistic regression for crude and adjusted analyses between the outcome and exposures. Variables with *p* ≤ 0.20 were included in the multivariate model, adjusted for confounding factors, and remained in the model with a *p*-value ≤0.05 (Figure 1). The results were presented as adjusted Odds Ratio (ORa) and 95% confidence intervals (95% CI). The analyses were performed using Stata 17.0 software, ensuring robustness in the identification of associated factors.

### 2.7 Analytical conceptual model

The adjustment of the data analysis followed the conceptual model presented in Figure 1, which was developed based on a literature review of factors associated with dating psychological violence. The model was structured hierarchically into three levels: Level 1 included sociodemographic characteristics; Level 2 comprised behavioral characteristics; and Level 3 included variables related to experiences of violence. This hierarchical approach contributed to the coherence of the analytical process, allowing distal variables to influence more proximal factors. Thus, the adjustment described in the model was performed considering the sequential inclusion of variables according to their respective levels in relation to the occurrence of dating psychological violence.

### 2.8 Ethical aspects

While respecting ethical considerations, the study was submitted to the Ethics and Research Committee of the Federal University of Espírito Santo and conducted after approval and registration under Opinion N°5.900.370.

## 3 Results

The results show that 18.6% (95% CI: 16.5–20.8) of 1,238 adolescents who were in a romantic relationship reported experiencing dating psychological violence. When analyzing the results by gender, we found that 21.7% (95% CI: 19.0–24.7) of girls reported experiencing this type of violence, while the prevalence was 13.1% (95% CI: 10.3–16.6) among boys (Table 1).

**Table 1:** Prevalence of dating psychological violence among high school students in the metropolitan region of Vitória, Espírito Santo, in 2023 (*N* = 1.238).

	General sample			Female			Male		
	*N*	%	95% CI	*N*	%	95% CI	*N*	%	95% CI
Dating psychological violence (*n* = 1,238)									
Yes	230	18.6	16.5–20.8	171	21.7	19.0–24.7	59	13.1	10.3–16.6
No	1,008	81.4	79.2–83.5	617	78.3	75.3–81.0	391	86.9	83.4–89.7

*N*, absolute frequency; %, relative frequency; CI, confidence interval.

Table 2 shows a relationship between dating psychological violence and the variables gender, cisgender, heterosexual, religion, sexual intercourse in lifetime, current tobacco use, current drug use, and current alcohol use, as well as between those who have suffered cyberbullying and those who have suffered and perpetrated bullying (*p* < 0.05).

**Table 2:** Distribution of psychological violence among high school adolescents in the Metropolitan Region of Vitória, Espírito Santo, in 2023, per socioeconomic and behavioral characteristics and violence experiences.

Variables	Psychological violence		
	Yes	95% CI	*p*-value
Sociodemographic			
Gender (*n* = 1.238)			<0.001
Female	171 (21.7)	19.0–24.7	
Male	59 (13.1)	10.3–16.6	
Age group (*n* = 1,238)			0.184
≤15 years	36 (15.5)	11.4–20.8	
≥16 years	194 (19.3)	16.1–21.8	
Cisgender (*n* = 1,237)			0.008
Yes	202 (17.7)	15.6–20.1	
No	28 (28.6)	20.5–38.3	
Heterosexual (*n* = 1,238)			<0.001
Yes	143 (15.2)	13.0–17.6	
No	87 (29.5)	24.6–35.0	
Ethnicity/skin color (*n* = 1,195)			0.571
White	93 (19.8)	16.4–23.6	
Black	40 (18.4)	13.8–24.1	
Brown	87 (17.2)	14.1–20.7	
ABEP (*n* = 1,237)			0.931
A	41 (18.6)	14.0–24.3	
B	101 (18.2)	15.2–21.6	
C/D/E	88 (19.1)	15.8–22.9	
Work/job (*n* = 1,238)			0.571
No	153 (18.2)	15.7–20.9	
Yes	77 (19.5)	15.9–23.7	
Religion (*n* = 1,238)			0.015
None	69 (22.3)	18.0–27.3	
Catholic	46 (15.3)	11.7–19.9	
Evangelical	86 (16.6)	13.6–20.1	
Other	29 (26.1)	18.8–35.1	
Behavioral			
Sexual intercourse in lifetime (*n* = 1,238)			<0.001
No	62 (13.0)	10.2–16.3	
Yes	168 (22.1)	19.3–25.2	
Current tobacco use (*n* = 402)			0.006
No	77 (22.8)	18.6–27.6	
Yes	25 (39.1)	27.9–51.5	
Current drug use (*n* = 1,238)			<0.001
No	183 (16.8)	14.7–19.2	
Yes	47 (31.3)	24.4–39.2	
Current alcohol use (*n* = 1,238)			<0.001
No	119 (15.3)	13.0–18.0	
Yes	111 (24.0)	20.3–28.1	
Violence experiences			
Suffered cyberbullying in lifetime (*n* = 1,238)			<0.001
No	193 (17.0)	14.9–19.3	
Yes	37 (37.0)	28.1–46.9	
Perpetrated cyberbullying in lifetime (*n* = 1,238)			0.107
No	213 (18.2)	16.1–20.5	
Yes	17 (26.2)	16.9–38.1	
Suffered bullying in lifetime (*n* = 1,171)			<0.001
No	60 (10.8)	8.5–13.6	
Yes	156 (25.4)	22.1–29.0	
Perpetrated bullying in lifetime (*n* = 1,238)			0.003
No	181 (17.2)	15.0–19.6	
Yes	49 (26.5)	20.6–33.3	

CI, confidence interval.

After controlling for confounding factors, we observed that girls were 1.53 times more likely to experience dating psychological violence (95% CI: 1.09–2.14). Similarly, those who identified as non-heterosexual were about twice as likely to experience this type of abuse (ORa: 2.09; 95% CI: 1.52–2.87). Those who reported having had sexual intercourse in their lifetime were 2.18 times more likely to suffer dating psychological violence (ORa: 2.18; 95% CI: 1.05–4.51), as were those who reported current tobacco use, who were more likely to experience this type of abuse in their relationship (ORa: 1.98; 95% CI: 1.11–3.54). Finally, adolescents who had experienced bullying in their lifetime were four times more likely to experience dating psychological violence (ORa: 4.11; 95% CI: 2.37–7.13) (Table 3).

**Table 3:** Crude and adjusted analysis of dating psychological violence among high school adolescents in the Metropolitan Region of Vitória, Espírito Santo, in 2023, associated with socioeconomic and behavioral characteristics and violence experiences.

Psychological violence						
Variables	OR	95% CI	*p*-value	ORa	95% CI	*p*-value
Sociodemographic						
Gender (*n* = 1,238)			<0.001			0.013
Female	1.84	1.33–2.53		1.53	1.09–2.14	
Male	1			1		
Age group (*n* = 1,238)			0.185			0.102
≤15 years	1			1		
≥16 years	1.30	0.88–1.92		1.39	0.94–2.06	
Cisgender (*n* = 1,237)			0.009			0.191
Yes	1			1		
No	1.86	1.17–2.95		1.39	0.85–2.27	
Heterosexual (*n* = 1,238)			<0.001			<0.001
Yes	1			1		
No	2.34	1.72–3.18		2.09	1.52–2.87	
Ethnicity/skin color (*n* = 1,195)			0.571			-
White	1			-	-	
Black	0.91	0.60–1.37		-	-	
Brown	0.84	0.61–1.16		-	-	
ABEP (*n* = 1,237)			0.931			-
A	1			-		
B	0.97	0.65–1.45		-	-	
C/D/E	1.03	0.68–1.55		-	-	
Work/job (*n* = 1,238)			0.571			-
No	1			-	-	
Yes	1.09	0.81–1.48		-	-	
Religion (*n* = 1,238)			0.016			0.543
None	1			1		
Catholic	0.63	0.42–0.95		0.78	0.51–1.20	
Evangelical	0.69	0.49–0.99		0.86	0.59–1.25	
Other	1.23	0.75–2.03		1.12	0.67–1.87	
Behavioral						
Sexual intercourse in lifetime (*n* = 1,238)			<0.001			0.036
No	1			1		
Yes	1.90	1.39–2.61		2.18	1.05–4.51	
Current tobacco use (*n* = 402)			0.007			0.021
No	1			1		
Yes	2.17	1.24–3.81		1.98	1.11–3.54	
Current drug use (*n* = 1,238)			<0.001			0.470
No	1			1		
Yes	2.26	1.54–3.30		1.23	0.71–2.13	
Current alcohol use (*n* = 1,238)			<0.001			0.125
No	1			1		
Yes	1.76	1.31–2.33		1.52	0.89–2.61	
Violence experiences						
Suffered cyberbullying in lifetime (*n* = 1,238)			<0.001			0.060
No	1			1		
Yes	2.88	1.86–4.44		2.10	0.97–4.54	
Perpetrated cyberbullying in lifetime (*n* = 1,238)			<0.001			0.960
No	1			1		
Yes	1.60	0.90–2.83		1.02	0.40–2.65	
Suffered bullying in lifetime (*n* = 1,171)			<0.001			<0.001
No	1			1		
Yes	2.82	2.04–3.90		4.11	2.37–7.13	
Perpetrated bullying in lifetime (*n* = 1,238)			0.003			0.456
No	1			1		
Yes	1.74	1.21–2.50		1.26	0.69–2.29	

CI, confidence interval; OR, crude odds ratio; ORa, adjusted odds ratio.

OR, crude odds ratio; ORa, adjusted odds ratio; CI, confidence interval. *p*-values obtained using logistic regression. The reference category corresponds to OR = 1.

## 4 Discussion

The prevalence of 18.6% (95% CI: 16.5–20.8) of dating psychological violence observed in this study indicates that this type of abuse is a common form of violence among adolescents, confirming previous findings that this type of violence is one of the most frequent manifestations in youth affective relationships (11). In a study conducted with 1,008 adolescents from six European countries, victimization by psychological/emotional violence showed an overall prevalence close to 30%, with 28.1% among girls and 21.0% among boys, revealing that controlling behaviors, humiliations, and intimidation are widely observed in youth affective relationships (12).

The higher prevalence of dating psychological violence among adolescents can be understood in light of the normalization of abusive practices, which leads to controlling or manipulative behaviors being seen as a “normal” part of the relationship. Brazilian studies show that many young people interpret excessive jealousy, constant criticism, or monitoring as legitimate demonstrations of affection, which favors the acceptance of these acts and reduces the perception of risk (5). This distorted understanding of the dynamics of intimacy creates an environment in which psychological violence takes hold subtly, without generating the unease that would accompany more explicit abusive forms. Thus, normalization directly contributes to this type of violence being frequent and socially tolerated among adolescents (1, 13).

Psychological violence’s invisibility is central to its high prevalence. Because it does not produce physical marks and involves ambiguous behaviors such as emotional blackmail, manipulation, veiled humiliations, or digital surveillance, this form of violence tends to be minimized by the adolescents themselves and even by professionals, families, and educational institutions (13). The difficulty in identifying signs of psychological abuse compromises the recognition of the situation of violence, delaying the search for support and reducing the likelihood of reporting, which reinforces the persistence and hinders the interruption of the psychological abuse cycle (1).

Among girls, the prevalence was 21.7%, while among boys it was 13.1%, meaning that the data shows that females are about 1.53 times more likely to suffer dating psychological violence. This finding is consistent with a study by Javier-Juárez et al. (11), which indicates that girls have higher dating psychological victimization rates. In a study conducted in Quebec, Canada, Pica et al. (14) identified that 26.6% of adolescent girls reported psychological violence experiences, while this prevalence was 16.9% among boys, indicating a substantial difference between genders. Similarly, research conducted in Spain with adolescents aged 13–17 showed that girls were more exposed to controlling and emotionally intimidating behaviors, with 22% reporting victimization, compared to 20.5% of boys (12).

This result can be primarily attributed to the influence of traditional gender roles and inequality in affective relationships, in which controlling and devaluing behaviors tend to be more frequently directed at girls. Research indicates that adolescents are socialized into norms that reinforce male authority and female emotional responsibility, favoring naturalized surveillance, jealousy, and emotional disqualification (15).

Furthermore, ambivalent sexism, as proposed by Glick and Fiske, helps to explain this situation: it combines hostile sexism, based on attitudes of criticism and domination, with benevolent sexism, which emerges as care and protection, but portrays girls as fragile and dependent. In simple terms, this means that attitudes that appear to be demonstrations of affection, such as “over-caring,” monitoring, excessive jealousy, or limiting friendships, can be perceived as natural or even positive, reinforcing male control and less female autonomy (16). Furthermore, traditional representations of masculinity, associated with dominance, and femininity, linked to passivity, reinforce the greater vulnerability of girls to this type of violence (17).

Another finding of the present study is the higher prevalence of dating psychological violence among adolescents who did not identify as heterosexual (ORa = 2.09; 95% CI: 1.52–2.87). This evidence is consistent with a systematic review by Sulla et al. (18), which identified a higher prevalence of dating violence among young people belonging to sexual and gender minorities, particularly psychological victimization. The authors also highlight that stigma, discrimination, and lack of social support can contribute to exposure to abusive situations.

Regarding behavioral factors, sexual initiation was associated with higher odds of dating psychological violence (ORa = 2.18; 95% CI: 1.05–4.51), as was current tobacco use (ORa = 1.98; 95% CI: 1.11–3.54). These findings are similar to those of Malherbe et al. (19), who observed an association between risky behaviors and dating violence experiences. According to these authors, adolescents who adopt behaviors such as tobacco use and early initiation of sexual activity are more prone to abusive relationships, since substance use can function as an element that increases vulnerability to dating violence and an unfolding of this very abusive experience, possibly due to impulsivity and difficulty in recognizing healthy boundaries in affective interactions (20).

Adolescents exposed to bullying were 4.11 (95% CI: 2.37–7.13) times more likely to experience dating psychological violence. Reviews conducted by Espelage et al. (21) showed that experiences of peer victimization are directly associated with a higher probability of involvement in abusive relationships. The authors emphasize that bullying can act as a precursor to violent dating behavior, as it promotes the normalization of abuse and compromises socio-emotional skills.

Exposure to bullying appears to open a pathway that increases the risk of dating psychological violence, which is why family and school are central in early detection, supervision, and intervention. Reviews and longitudinal studies suggest that peer victimization experiences contribute to the normalization of abuse and impair essential socio-emotional skills, making adolescents more vulnerable to abusive relationships (21, 22). In this sense, parenting practices that include consistent supervision, open communication, and nonviolent discipline act as protective factors by reducing risky behaviors and improving the emotional regulation of adolescents (22).

Dating psychological violence has emerged as one of the most harmful forms of victimization in adolescence, as it produces direct and persistent impacts on adolescents’ mental health. Recent evidence shows that humiliation, control, insults, and intimidation within romantic relationships are strongly associated with increased depressive symptoms, anxiety, suicidal ideation, and poorer emotional functioning over time (23). Psychological violence is the most prevalent form of abuse in dating relationships and is related to low self-esteem, high emotional reactivity, and greater vulnerability to future abusive relationships, reinforcing its clinical and social relevance (24). Due to the higher risks of internalizing problems and revictimization, we underscore the need for effective preventive strategies aimed at this type of violence (25).

In the school context, universal interventions, especially programs based on a whole-school approach, promotion of socio-emotional skills, and strengthening of a supportive climate, have reduced bullying and dating violence indicators when applied in an integrated and continuous manner (26, 27, 33). Systematic reviews on socioemotional learning further reinforce that improving skills such as empathy, self-control, and conflict resolution decreases the acceptance of aggressive behaviors and improves the quality of peer interactions (28).

However, the findings of this study suggest that prevention efforts should also address broader structural and contextual factors within schools. Evidence indicates that school climate plays a critical role in shaping adolescents’ relationship norms, as students who perceive their environment as safe and supportive are less likely to accept or perpetrate violence in their romantic relationships (29). Conversely, schools with high levels of peer aggression may inadvertently reinforce the normalization of controlling behaviors among adolescents (30). Therefore, comprehensive strategies should integrate clear anti-violence policies, continuous teacher training to recognize signs of psychological abuse, accessible support services for victims, and active engagement of families and the community in reinforcing healthy relationship models (31). Such multilevel approaches, which address individual, relational, and institutional factors simultaneously, have shown greater effectiveness in reducing dating violence than isolated programs focused solely on student skills (29).

This study has some limitations. Since it only covers adolescents attending school, it does not include dropouts, who may display different, and possibly more pronounced, dating psychological violence patterns. Furthermore, as it is a cross-sectional study, the results correspond to a single observation point in time, which prevents the identification of causal relationships and may involve recall bias. Despite these limitations, the study offers relevant contributions to the understanding of psychological violence in the context of relationships among school-aged adolescents. It is also the first to present representativeness for young people from a large Brazilian metropolitan region.

## 5 Conclusion

The prevalence of 18.6% of dating psychological violence identified among adolescents in this study indicates that emotional abuse and controlling behaviors are widely present in youth affective relationships. This type of abuse was associated with female sex, non-heterosexual orientation, history of sexual intercourse, current cigarette use, and previous experiences of bullying. These results reveal that behaviors such as humiliation, intimidation, and manipulation emerge in early romantic experiences, potentially compromising socioemotional development during this stage of life.

These findings reinforce that psychological victimization is linked both to social vulnerabilities and to experiences of violence in other contexts, suggesting a cycle of aggression that permeates different dimensions of adolescents’ lives. This evidence is highly relevant for health, education, and social assistance professionals, as well as for public managers, as it contributes to guiding prevention and intervention strategies in adolescents’ affective relationships.

Furthermore, the findings highlight the need to promote educational actions that address not only the recognition of abusive behaviors but also mutual respect, healthy communication, and the development of safe relationships. The active involvement of families and schools may enhance the reach of these initiatives, supporting the early identification of signs of psychological violence and the provision of qualified support to victims. In addition, the association between bullying and dating psychological violence reinforces the urgency of integrated public policies that consider the interrelation between different forms of violence affecting adolescents’ school and affective lives. Similarly, the relationship with cigarette use and sexual initiation suggests the need for broader approaches that simultaneously address mental health, risk behaviors, and education for healthy relationships.

In this context, the results of this study reiterate the importance of multidisciplinary actions aimed at preventing dating psychological violence, focusing on reducing risk factors and strengthening protective factors. Promoting supportive school environments, strengthening family bonds, and encouraging youth empowerment are essential measures for building safer, more respectful, and equitable affective relationships.

## Data Availability

The raw data supporting the conclusions of this article will be made available by the authors, without undue reservation.
